# Sex-specific in utero reprogramming of lung immunity

**DOI:** 10.1016/j.mucimm.2026.100352

**Published:** 2026-06-01

**Authors:** Anthony Maxwell, Audrey Couturier, Annie Thy Nguyen, Savannah Schick, Aditi Singh, Elizabeth Findeis, Jayanth Ramadoss, Judy Westrick, Nicholas Peraino, Paul M. Stemmer, Albert M. Levin, Gil Mor, Jiahui Ding

**Affiliations:** a C.S Mott center for Human Growth and Development, Department of Obstetrics and Gynecology, Wayne State University, Detroit, MI, USA; b Department of Biochemistry, Microbiology and Immunology, Wayne State University, Detroit, MI, USA; c School of Medicine, Wayne State University, Detroit, MI, USA; d Department of Chemistry, Lumigen Instrumentation Center, Wayne State University, Detroit, MI, USA; e Institute of Environmental Health Sciences and Department of Pharmacology, Wayne State University, Detroit, MI, USA; f Department of Public Health Sciences, Henry Ford Health, Detroit, MI, USA; g Center for Bioinformatics, Henry Ford Health, Detroit, MI, USA

**Keywords:** Prenatal exposure, Sex differences, Lung immunity, Interferon signaling, Alveolar macrophages, DREAM-A20

## Abstract

Lung mucosal immunity must balance effective antimicrobial defense with tightly controlled inflammatory responses to maintain pulmonary homeostasis. Although sex differences in respiratory disease susceptibility are well documented, the developmental origins of these differences and their modulation by prenatal environmental exposures remain poorly defined. Here, we tested the hypothesis that prenatal benzene exposure establishes sex-specific reprogramming of lung mucosal immunity during fetal development. Using a controlled inhalation exposure model in pregnant C57BL/6 mice, we characterized immune responses in fetal and postnatal lungs under baseline conditions and following viral challenge. We found that female offspring displayed heightened type I interferon signaling and enhanced viral clearance, accompanied by exaggerated inflammatory pathology and altered expressions of DREAM and A20. In contrast, male offspring exhibited augmented proinflammatory cytokine production following lipopolysaccharide challenge. Alveolar macrophages from prenatally benzene exposed offspring demonstrated persistent inflammatory priming, indicating durable alteration of innate immune populations. These findings demonstrate that lung mucosal immune development is intrinsically sex-dependent and that prenatal environmental pollutants interact with fetal sex to durably reprogram respiratory immunity. Together, our results identify in utero environmental exposure as a critical determinant of sex-specific mucosal immune trajectories that shape postnatal responses to infection.

## Introduction

The lung is a highly specialized mucosal organ that continuously interfaces with the external environment while maintaining the delicate physiological requirements of gas exchange.^1,2^ Lung mucosal immunity is therefore uniquely orchestrated: it must provide effective antimicrobial defense while maintaining control of inflammation to preserve tissue integrity.^1^ This balance is achieved through coordinated interactions between the epithelial barrier, resident innate immune populations, and recruited adaptive immune cells.^2,3^ Perturbation of this mucosal immune equilibrium contributes to susceptibility to infection and inflammatory lung disease,^3^ underscoring the central role of mucosal immune regulation in respiratory health.

Lung mucosal immunity is established through a tightly orchestrated developmental program that begins during gestation and continues to mature after birth. Innate immune cells, including macrophages, dendritic cells, and natural killer (NK) cells, seed the fetal lung early in development, coincident with epithelial differentiation and the emergence of antimicrobial defenses. These observations indicate that fundamental components of pulmonary mucosal immune surveillance and inflammatory regulation are established in utero.^4,5^ Following birth, lung immune maturation is further shaped by microbial colonization and environmental antigen exposure. Thus, lung mucosal immunity reflects the integration of prenatal immune programming with postnatal environmental exposures. Despite recognition that pulmonary mucosal immunity originates before birth, the extent to which pregnancy actively shapes lung immune development remains poorly defined. The intrauterine environment exposes the developing lung to maternal-derived cytokines, hormones, metabolic signals, and environmental factors, all of which have the potential to durably imprint fetal immune cells.^4,5^ Pregnancy therefore represents a critical window for the establishment of lung immune set points that may influence postnatal immune function and disease susceptibility.^6–8^ However, the mechanisms by which maternal and placental signals regulate the differentiation and functional programming of lung mucosal immune populations remain poorly understood.

Importantly, lung development and immune regulation are strongly influenced by sex as a biological variable. Respiratory diseases exhibit marked sexual dimorphism across the life course, with sex-dependent differences in incidence, severity, and inflammatory phenotype.^9–11^ These differences are not solely attributable to postnatal hormonal effects but instead reflect developmental origins that precede birth. Within this developmental framework, environmental exposures during pregnancy represent potent modulators of lung mucosal immune programming. The fetal lung is particularly vulnerable to such perturbations, as immune cells seed the mucosa early in gestation and are capable of sensing inflammatory and toxicant-derived signals in utero.^4,5^ Epidemiological and experimental studies demonstrate that maternal exposure to air pollutants, tobacco smoke, and other environmental stressors alters fetal lung development^12–15^ and increases offspring susceptibility to respiratory disease.^16–18^ Importantly, these exposures induce sex-specific transcriptional and immunological changes in the developing lung, suggesting that environmental exposure triggers sex-specific responses to establish divergent mucosal immune trajectories. Mechanistic studies implicate multiple pathways in this environmentally driven, sex-specific lung immune programming, including sex-dependent placental immune and endocrine signalling,^19,20^ stress hormone signaling through the hypothalamic–pituitary–adrenal axis,^21^ epigenetic modifications,^22,23^ and early programming of lung resident immune cells.^24,25^ However, how these processes converge to shape antiviral and inflammatory responses in the postnatal lung remains poorly defined.

Here, we investigated whether prenatal exposure to a human-relevant environmental pollutant establishes sex-specific lung mucosal immune programming that persists beyond birth and shapes postnatal responses to infection. Using gestational benzene exposure in mice, we analyzed immune development and inflammatory responses in both fetal and postnatal lungs across sex and secondary immune challenge. We identify significant, intrinsic differences in lung mucosal immune development between males and females that are evident during fetal life, demonstrating that sexual dimorphism in pulmonary mucosal immunity is established in utero. In addition to these baseline developmental differences, prenatal benzene exposure exerts strongly sex-specific effects on lung immune programming, inducing durable and differential immune trajectories in male and female offspring. Functionally, these sex-dependent programs result in distinct antiviral and inflammatory outcomes after birth. Mechanistically, our data implicate altered DREAM–A20 regulatory dynamics as a potential female-biased pathway associated with interferon hyperactivation and inflammatory imbalance following prenatal benzene exposure. Together, our findings demonstrate that lung mucosal immune development is intrinsically sex-dependent and that prenatal environmental pollutants interact with fetal sex to durably reprogram respiratory immunity, thereby shaping postnatal susceptibility to infections.

## Results

### Sexual dimorphism in pulmonary antiviral responses to viral infection

**Fig. 1.**
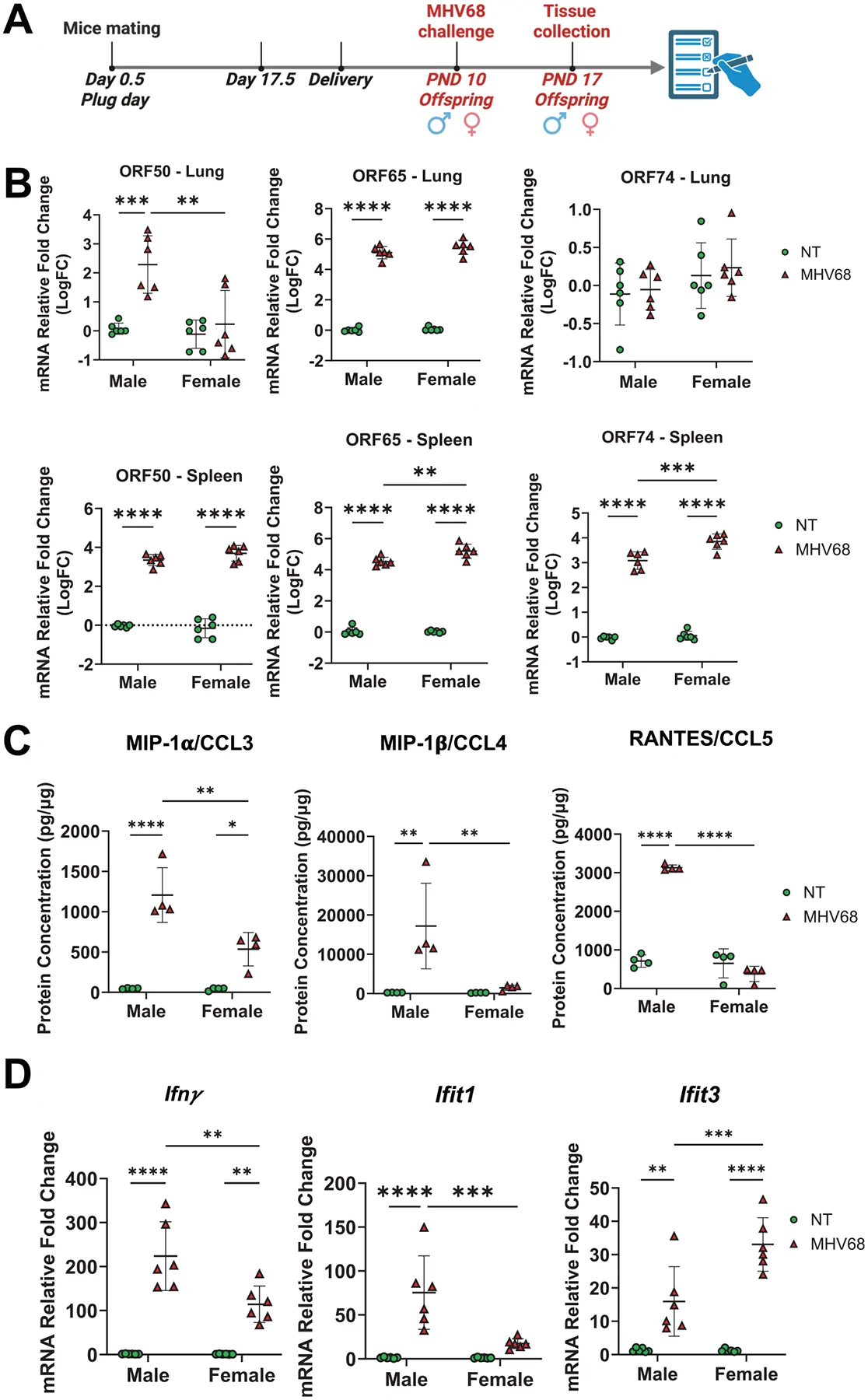
Sex-dimorphic pulmonary and systemic immune responses to MHV68 infection in neonatal mice. (A) Experimental breeding and infection scheme. Plug-positive C57BL/6 dams were time-mated to generate offspring, which were challenged with MHV68 (65 PFU, i.p.) at postnatal day 10 (PND10) and harvested 7 days post-infection for downstream analyses. Created in BioRender. (B) Viral burden in PND17 lung and spleen determined by qRT-PCR for ORF50 (early lytic), ORF65 (late lytic), and ORF74 (latency-associated) viral genes. Data are presented as log10-transformed copy numbers normalized to Ppia expression. For each group, n = 6 offspring were analyzed, representing 2 independent dams/litters with 3 offspring sampled from each dam/litter. (C) Cytokine and chemokine levels in PND17 lung protein lysates measured by Luminex multiplex assay. For each group, n = 4 offspring were analyzed, representing 2 independent dams/litters with 2 offspring sampled from each dam/litter. (D) qRT-PCR analysis of IFN-γ and interferon-stimulated genes (ISGs) (Ifit1 and Ifit3) in PND17 lungs following MHV68 infection. Expression levels were normalized to Ppia. For each group, n = 6 offspring were analyzed, representing 2 independent dams/litters with 3 offspring sampled from each dam/litter. Data are presented as mean ± SD; statistical comparisons were performed using two-way ANOVA with Tukey’s post hoc test where appropriate. *P <* 0.05 (*), P < 0.01 (**), P < 0.001 (***), P < 0.0001 (****).

To assess sex difference in neonatal pulmonary antiviral immunity, C57BL/6 pups were challenged with murine gammaherpesvirus 68 (MHV68, 65 PFU, i.p.) at postnatal day 10 (PND10) and tissues were collected at PND17 (Fig. 1A). MHV68 is a mouse herpesvirus that efficiently replicates in the lung epithelium and represents a murine model of respiratory viral infection.^26^ MHV68 viral gene expressions representing early lytic (*Orf50*), late lytic (*Orf65*), and latency (*Orf74*) stages^26^ were quantified by qRT-PCR. In the lung, *Orf50* expression was significantly higher in infected males than females, indicating greater early lytic activity in males. *Orf65* expression increased with infection but did not differ between sexes. *Orf74* was not elevated during infection in either sex in the lung at this timepoint (Fig. 1B). In the spleen, *Orf50* expression was elevated in both sex without difference; however, *Orf65* and *Orf74* were significantly higher in infected females than males (Fig. 1B). These findings reveal organ- and sex-specific differences in viral replication, with enhanced early lytic activity in male lungs and greater late-stage and latency-associated viral expression in female spleens.

We next evaluated inflammatory responses in lung homogenates using Luminex. CCL3, CCL4, and CCL5 were significantly elevated in infected male lungs than in females (Fig. 1C). Consistent with this pattern, *Ifn*-*γ* and *Ifit1* expression were significantly higher in infected male lungs, whereas *Ifit3* expression was selectively increased in infected female lungs (Fig. 1D). Collectively, these results reveal robust sexual dimorphism in neonatal antiviral responses to MHV68 infection, reflecting organ-specific and viral stage-dependent regulation of anti-viral defense.

### Gestational benzene exposure alters sex-specific viral replication in neonates

**Fig. 2.**
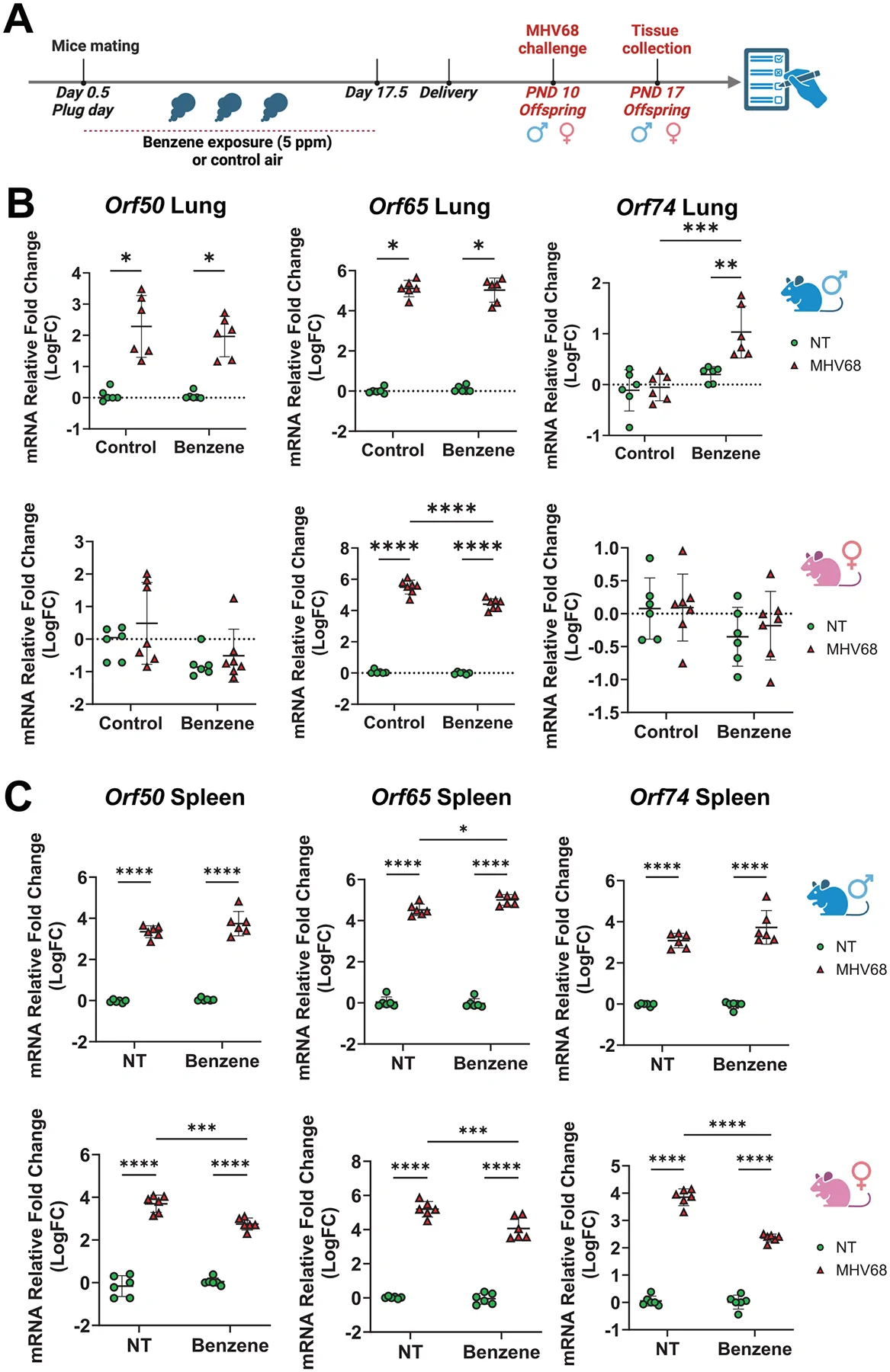
Sex-dimorphic MHV68 replication in the lung and spleen following gestational benzene exposure. (A) Experimental design. Pregnant dams were exposed to benzene vapor (5 ppm) from E0.5–E17.5, then transferred to a clean-air environment for delivery. Offspring were maintained under unexposed conditions until PND10. At PND10, pups were infected intraperitoneally with MHV68 (65 PFU) and sacrificed seven days later (PND17) for immune analysis. Created in BioRender. (B) Lung and spleen viral burden in PND17 offspring determined by qRT-PCR for ORF50, ORF65, and ORF74. Data are presented as log10-transformed copy numbers normalized to Ppia expression. For each group, n = 6 offspring were analyzed, representing 2 independent dams/litters with 3 offspring sampled from each dam/litter. (C) Spleen viral burden in PND17 offspring determined by qRT-PCR for ORF50, ORF65, and ORF74. Data are presented as log10-transformed copy numbers normalized to Ppia expression. For each group, n = 6 offspring were analyzed, representing 2 independent dams/litters with 3 offspring sampled from each dam/litter. Data are presented as mean ± SD; statistical comparisons were performed using two-way ANOVA with Tukey’s post hoc test where appropriate. *P <* 0.05 (*), P < 0.01 (**), P < 0.001 (***), P < 0.0001 (****).

To determine whether gestational benzene exposure modifies neonatal antiviral responses, plug-positive C57BL/6 dams were exposed to 5 ppm benzene or clean air from embryonic day 0.5 (E0.5) to E17.5 using inhalation chambers, then transferred to a clean air environment and allowed to deliver naturally (Fig. 2A). Offspring were maintained under clean conditions and challenged with MHV68 (65 PFU, i.p.) at PND10 and analyzed after 7 days.

In male lungs, *Orf50* and *Orf65* were significantly induced by infection regardless of prenatal exposure, with no significant differences between groups (Fig. 2B). In contrast, *Orf74* expression was detectable only in infected males born to benzene-exposed dams, indicating that gestational benzene exposure selectively promotes a latency-associated viral signature in the male lung. In females, *Orf50* was not increased in either group. *Orf65* increased following infection but was significantly reduced in female offspring from benzene-exposed dams compared with infected control offspring. *Orf74* was not increased in either exposure group in the female lung following infection (Fig. 2B). Together, these data indicate that gestational benzene exposure modifies the viral replication process in the offspring lung in a sex-dimorphic manner.

Because MHV68 disseminates systemically and establishes infection in lymphoid tissues, we assessed viral gene expression in the spleen by qRT-PCR. In male offspring, *Orf50* and *Orf74* was significantly increased following infection in both control and benzene-exposed groups without differences; however*, Orf65* was significantly higher in offspring from benzene-exposed dams (Fig. 2C), indicating enhanced late lytic activity modified by gestational benzene exposure. In female offspring, *Orf50*, *Orf65*, and *Orf74* were robustly induced in the spleen following infection in both exposure groups (Fig. 2C). Notably, female offspring from benzene-exposed dams showed significantly reduced expression of all three viral markers compared with infected control female offspring (Fig. 2C), suggesting improved viral suppression following gestational benzene exposure. Collectively, these findings demonstrate that gestational benzene exposure alters offspring response to MHV68 infection in a sex-dependent and organ-specific manner.

### Gestational benzene exposure amplifies sex-specific lung inflammation and interferon signaling during neonatal MHV68 infection

**Fig. 3.**
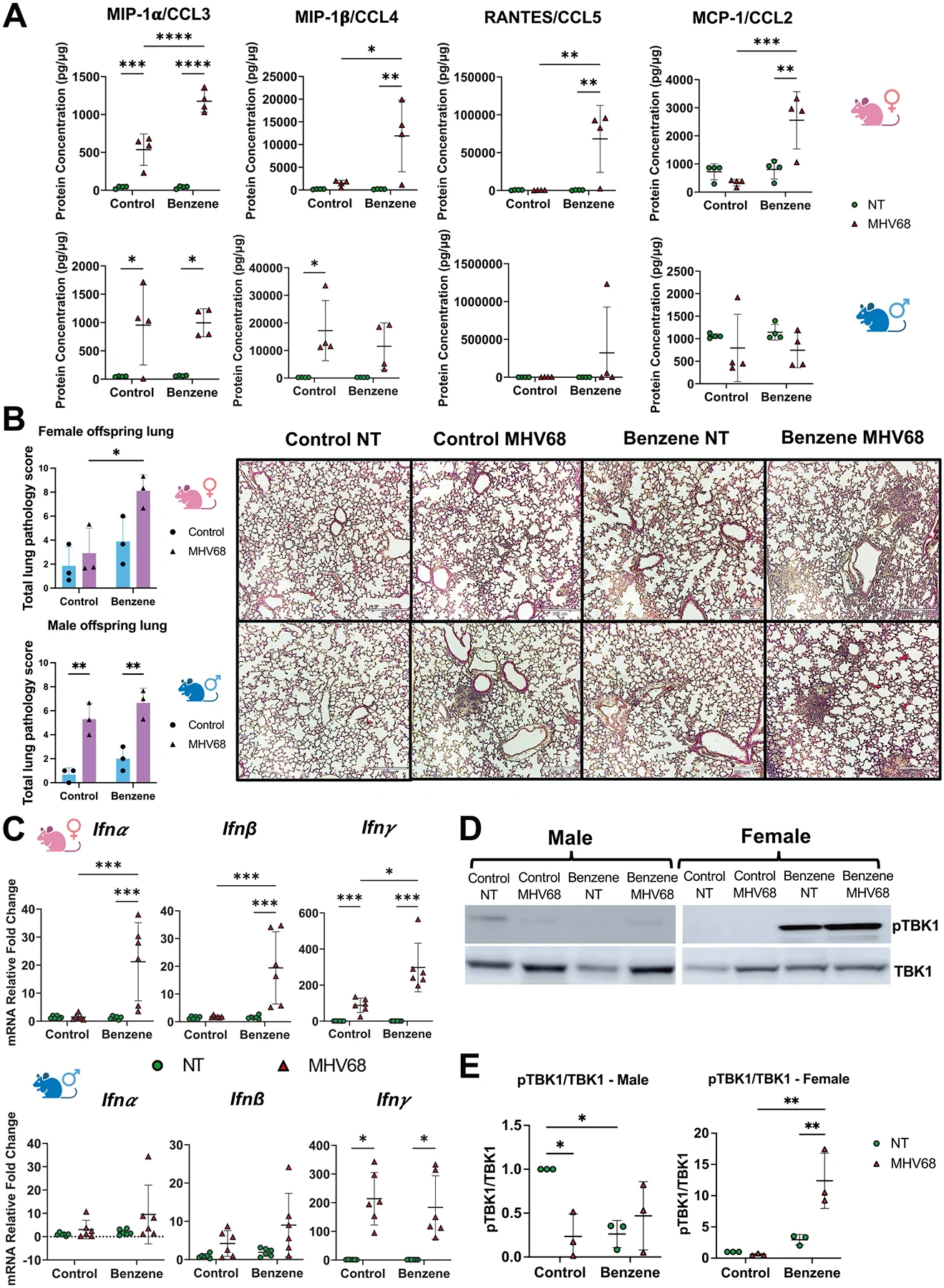
Sex-dimorphic tissue inflammation and interferon signaling in the neonatal lung following MHV68 infection. Offspring from control and gestational benzene-exposed dams were challenged with MHV68 at PND10 and lungs were harvested 7 days post-infection for inflammatory, histologic, and interferon signaling analyses. (A) Lung chemokine protein abundance measured by multiplex Luminex in whole lung homogenates. For each group, n = 4 offspring were analyzed, representing 2 independent dams/litters with 2 offspring sampled from each dam/litter. (B) Representative hematoxylin and eosin (H&E)-stained lung sections from PND17 offspring and quantification of lung pathology score. For each group, n = 3 offspring were analyzed, representing 3 independent dams/litters with 1 offspring sampled from each dam/litter. (C) qRT-PCR analysis of interferon genes (Ifnα, Ifnβ, and Ifnγ) in PND17 lungs following MHV68 infection. Expression levels were normalized to Ppia. For each group, n = 6 offspring were analyzed, representing 2 independent dams/litters with 3 offspring sampled from each dam/litter. (D) Representative western blots of phosphorylated TBK1 (pTBK1), total TBK1, and GAPDH in PND17 lungs. (E) Quantification of relative pTBK1 and total TBK1 protein expression by ImageJ and pTBK1 to TBK1 ratio. For each group, n = 3 offspring were analyzed, representing 3 independent dams/litters with 1 offspring sampled from each dam/litter. Data are presented as mean ± SD; statistical comparisons were performed using two-way ANOVA with Tukey’s post hoc test where appropriate. *P <* 0.05 (*), P < 0.01 (**), P < 0.001 (***), P < 0.0001 (****).

Given the sex-specific viral gene replication observed in offspring following gestational benzene exposure, we next sought to determine whether viral-induced inflammation was effectively controlled. Luminex analysis of 23 cytokines and chemokines in lung protein lysates revealed that MCP-1, MIP-1α, MIP-1β, and RANTES were significantly elevated in the lungs of female offspring from benzene-exposed dams compared with controls following MHV68 infection, however this effect was not observed in male offspring (Fig. 3A). Consistent with these inflammatory changes, histopathological analysis demonstrated exacerbated lung injury in female offspring from benzene exposed dams during infection (Fig. 3B). Pathology was characterized by alveolar collapse, septal thickening, bronchiolar wall thickening, and hemorrhage. In contrast, infected male offspring displayed increased pathology scores regardless of gestational exposure status, indicating that prenatal benzene exposure selectively exacerbates infection-induced lung injury in females.

We next assessed the gene expression of interferons and anti-viral ISGs in the offspring lungs. As expected, type I interferons (*Ifnα* and *Ifnβ*) were not induced in infected controls, whereas type II interferon (*Ifnγ*) was significantly upregulated following MHV68 infection (Fig. 3C). This pattern is consistent with the known capacity of MHV68 to suppress type I interferon signaling and instead be controlled primarily through the type II interferon pathway.^27^ ISGs, including IFIT1, IFIT2, and IFIT3, form a functional antiviral complex that plays a critical role in the innate immune response,^28^ which were significantly upregulated in infected controls (Supplementary Fig. S1). Importantly, gestational benzene exposure enhanced interferon and ISGs signaling in female offspring. *Ifnα* and *Ifnβ* were significantly upregulated, accompanied by even higher induction of *Ifnγ* and *Ifits (ifit1, 2, 3)*, whereas these enhancement were not observed in male offspring. This sex-specific interferon activation may explain the better viral control in females following gestational benzene exposure (Fig. 2).

Because MHV68 suppresses type I interferon signaling by blocking TBK1 phosphorylation, a key step in interferon pathway activation,^29^ we next examined TBK1 activation in offspring lungs. Baseline phosphorylated TBK1 (pTBK1) levels were reduced in male offspring from benzene-exposed dams but elevated in female offspring from benzene-exposed dams relative to same-sex controls (Fig. 3D–E). Following infection, robust pTBK1 induction was observed exclusively in female offspring from benzene-exposed dams. Together, these findings demonstrate that gestational benzene exposure programs a female-specific enhancement of TBK1–interferon signaling during neonatal MHV68 infection.

### Gestational benzene exposure induces persistent, sex-dimorphic changes in the DREAM–A20 axis in fetal and offspring lungs

**Fig. 4.**
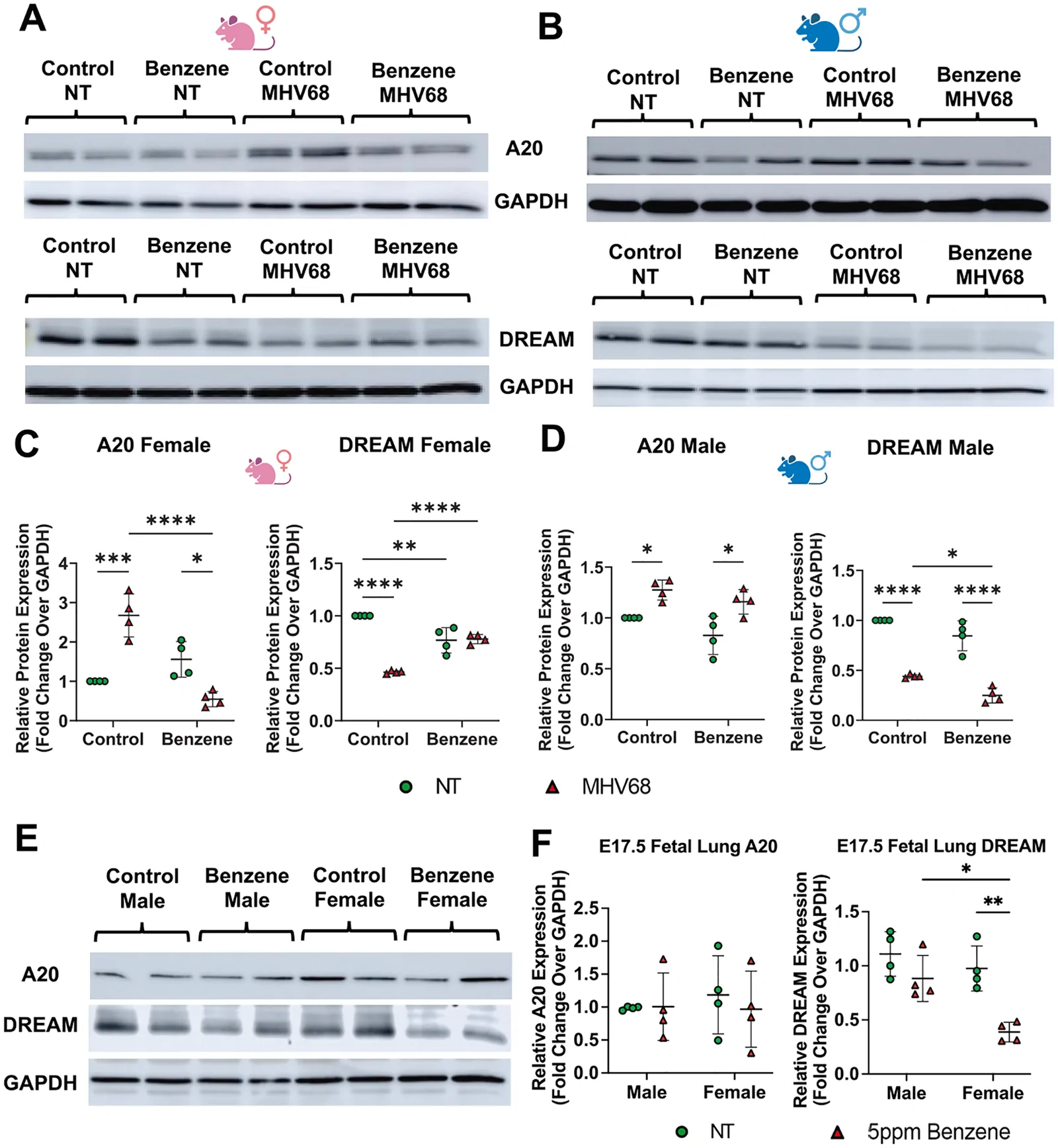
The DREAM–A20 regulatory axis is disrupted in fetal and neonatal lungs following gestational benzene exposure. (A,B) Representative western blots of A20, DREAM and GAPDH in PND17 lungs following neonatal MHV-68 infection. (C,D) Quantification of relative protein expression by ImageJ within each sex. For each group, n = 4 offspring were analyzed, representing 2 independent dams/litters with 2 offspring sampled from each dam/litter. (E) Representative western blots of A20, DREAM and GAPDH in E17.5 fetal lungs following maternal benzene exposure. (F) Quantification of relative protein expression by ImageJ. For each group, n = 4 offspring were analyzed, representing 2 independent dams/litters with 2 offspring sampled from each dam/litter. Data are presented as mean ± SD; statistical comparisons were performed using two-way ANOVA with Tukey’s post hoc test where appropriate. *P <* 0.05 (*), P < 0.01 (**), P < 0.001 (***).

Having established that TBK1–interferon signaling is aberrantly activated in the lungs of female offspring following gestational benzene exposure, we examined upstream negative regulators of this pathway. TNFAIP3 (A20) is a ubiquitin-editing enzyme that restrains NF-κB signaling and modulates type I interferon signaling via TBK1.^30^ DREAM, a calcium-dependent transcriptional repressor, controls the expression of Tnfaip3.^31^ The DREAM–A20 axis constitutes a key inhibitory mechanism that limits TBK1–interferon pathway activation. In the normal antiviral response, DREAM expression decreases while A20 is upregulated to control inflammation and interferon signaling, as observed in female control offspring following MHV68 infection. In male controls, A20 expression showed a trend toward increase but did not reach significance, suggesting a temporally delayed induction (Fig. 4A–D). Interestingly, gestational benzene exposure disrupted the DREAM–A20 axis specifically in female offspring lungs: females from benzene-exposed dams failed to downregulate DREAM and upregulate A20. Notably, DREAM was already reduced at baseline in females from benzene-exposed dams, potentially limiting further repression upon infection and contributing to impaired negative regulation of the TBK1–interferon pathway (Fig. 4A–D). Furthermore, western blot analysis revealed that female fetal lungs from benzene-exposed dams showed significantly reduced DREAM expression compared with controls (Fig. 4E, F). These findings suggest that DREAM may function as a female-specific developmental checkpoint that is disrupted by gestational benzene exposure.

### Gestational benzene exposure elevates placenta and fetal benzene metabolite levels and induces sex-specific inflammation

**Fig. 5.**
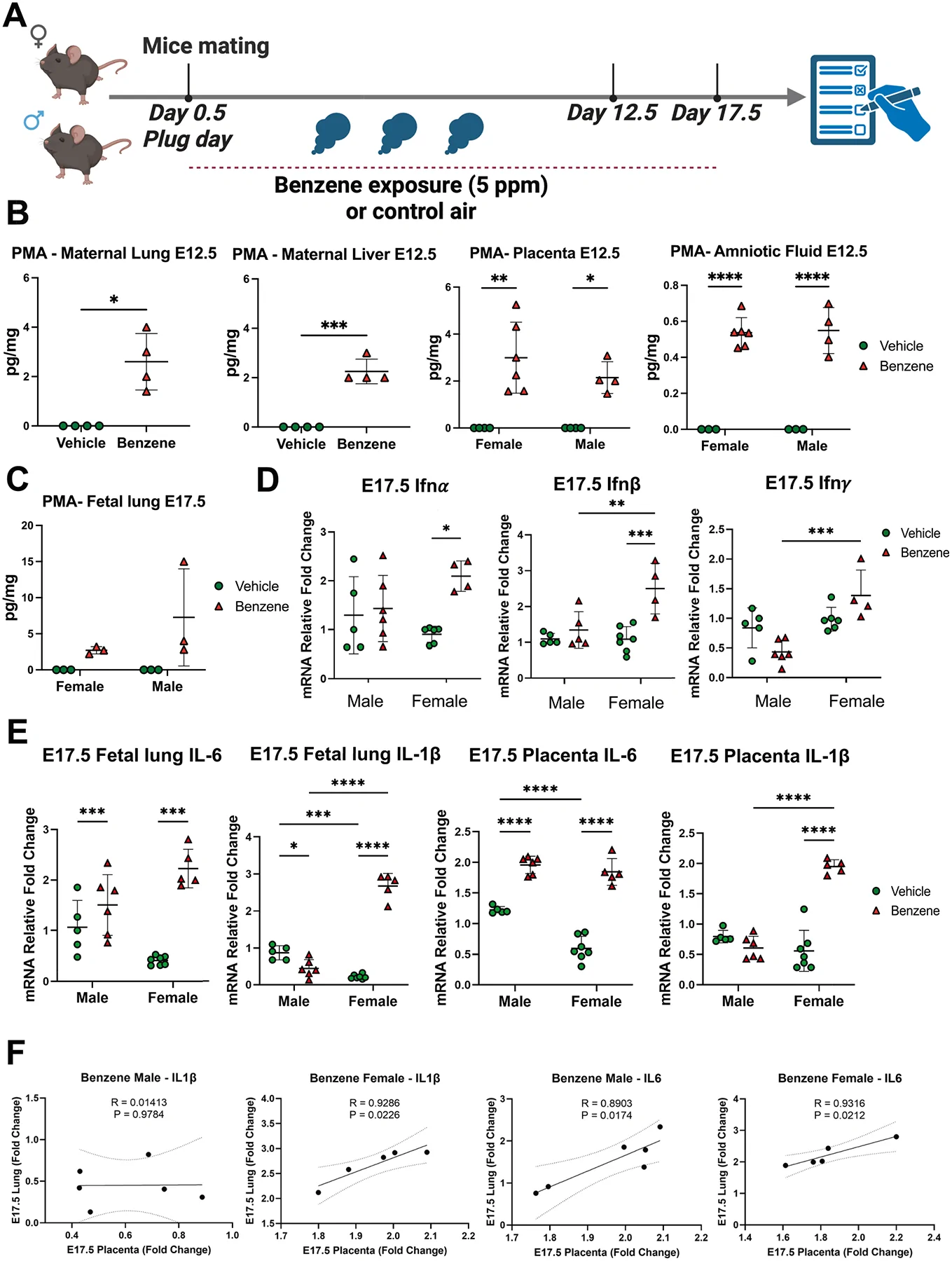
Sex-specific fetal lung inflammation following gestational benzene exposure. (A) Schematic of the experimental design showing maternal benzene inhalation mouse model (5 ppm, E0.5 to E17.5). Created in BioRender. (B) LC-MS quantification of the benzene metabolite PMA in maternal lung, liver, placenta, and amniotic fluid. Maternal lung and liver samples represent independent dams (n = 4 dams per group). Placenta and amniotic fluid samples were collected from fetuses derived from 3 independent dams/litters per group (n = 4 vehicle placentas; n = 6 benzene-exposed female placentas; n = 4 benzene-exposed male placentas; n = 3 vehicle amniotic fluid samples; n = 6 benzene-exposed female amniotic fluid samples; n = 4 benzene-exposed male amniotic fluid samples). (C) PMA concentrations in fetal lung confirm direct fetal exposure to benzene metabolites. Fetal lung samples were collected from 3 independent dams/litters per group (n = 3 per sex). (D) qRT-PCR analysis shows increased expression of type I and type II interferons (*Ifnα*, *Ifnβ*, and *Ifnγ)* in E17.5 fetal lungs from benzene-exposed dams. Fetal tissues were collected from 3 independent dams/litters per group (*n* = 5 vehicle males, 5 vehicle females, 6 benzene males, 4 benzene females.) (E) qRT-PCR analysis of pro-inflammatory genes (*Il6*, *Il1β*) reveals elevated expression in both placenta and fetal lung following 5 ppm benzene exposure. Gene expression normalized to *Ppia*. Fetal lung and placenta samples were collected from 3 independent dams/litters per group (*n* = 5 vehicle males, 5 vehicle females, 6 benzene males, 5 benzene females for fetal lung; 5 vehicle males, 6 vehicle females, 6 benzene males, 5 benzene females for placenta.) (F) Pearson Correlation analysis of *Il6* and *Il1β* expression across matched placenta–fetal lung pairs demonstrates coordinated inflammatory signaling at the maternal–fetal interface. Each point represents one placenta–fetal unit collected from 3 independent dams/litters per group (n = 5 pairs). Data are presented as mean ± SD; statistical comparisons were performed using unpaired *t* tests or two-way ANOVA with Tukey’s post hoc test where appropriate. *P <* 0.05 (*), P < 0.01 (**), P < 0.001 (***), P < 0.0001 (****).

Given postnatal lung immune alterations and reduced fetal DREAM expression, we next investigated whether fetal lungs are directly exposed and reprogrammed during gestation. Plug-positive C57BL/6 dams were exposed to 5 ppm benzene or control air continuously from E0.5 to E12.5 or E17.5, and maternal and fetal tissues were collected for downstream analysis (Fig. 5A). At E17.5, gestational benzene exposure did not broadly alter implantation number, resorption number, pregnancy loss rate, or fetal weight, although placental weight was reduced in male benzene-exposed fetuses (Supplementary Fig. S2). Phenylmercapturic acid (PMA), a gold-standard benzene metabolite^32^, was detected in maternal lungs and livers at E12.5, confirming effective exposure (Fig. 5B). PMA were also significantly elevated in E12.5 placentas and AF but did not differ by fetal sex (Fig. 5B). Since the size of the lungs at E12.5 is very small and difficult to dissect, we collected fetal lungs at E17.5 and detected PMA, with no sex-specific difference (Fig. 5C). These data indicate that in our mouse model, benzene metabolite PMA can reach the placenta and fetal lung.

We then assessed inflammatory signaling in E17.5 fetal lungs. Gestational benzene exposure induced significant female-specific increase of *Ifnα*, *Ifnβ*, and *Ifnγ*, whereas male fetal lungs showed no such increase (Fig. 5D). *Il6* expression was significantly upregulated in both sexes following gestational benzene exposure, with no sex difference (Fig. 5E). In contrast, *Il1b* increased only in female fetal lungs but decreased in males (Fig. 5E). Notably, baseline *Il1b* expression was higher in male fetal lungs compared to females. The placenta showed a similar trend for both cytokines (Fig. 5E). Correlation analysis revealed a strong positive association between placental and fetal lung *Il6* expression in both sexes, whereas *Il1b* expression correlated significantly only in females (Fig. 5F). Together, these results indicate that maternal benzene metabolites can reach the fetal lungs and induce sex-specific inflammatory responses during development.

### Persistent sex-specific transcriptomic changes in the developing lung following gestational benzene exposure

**Fig. 6.**
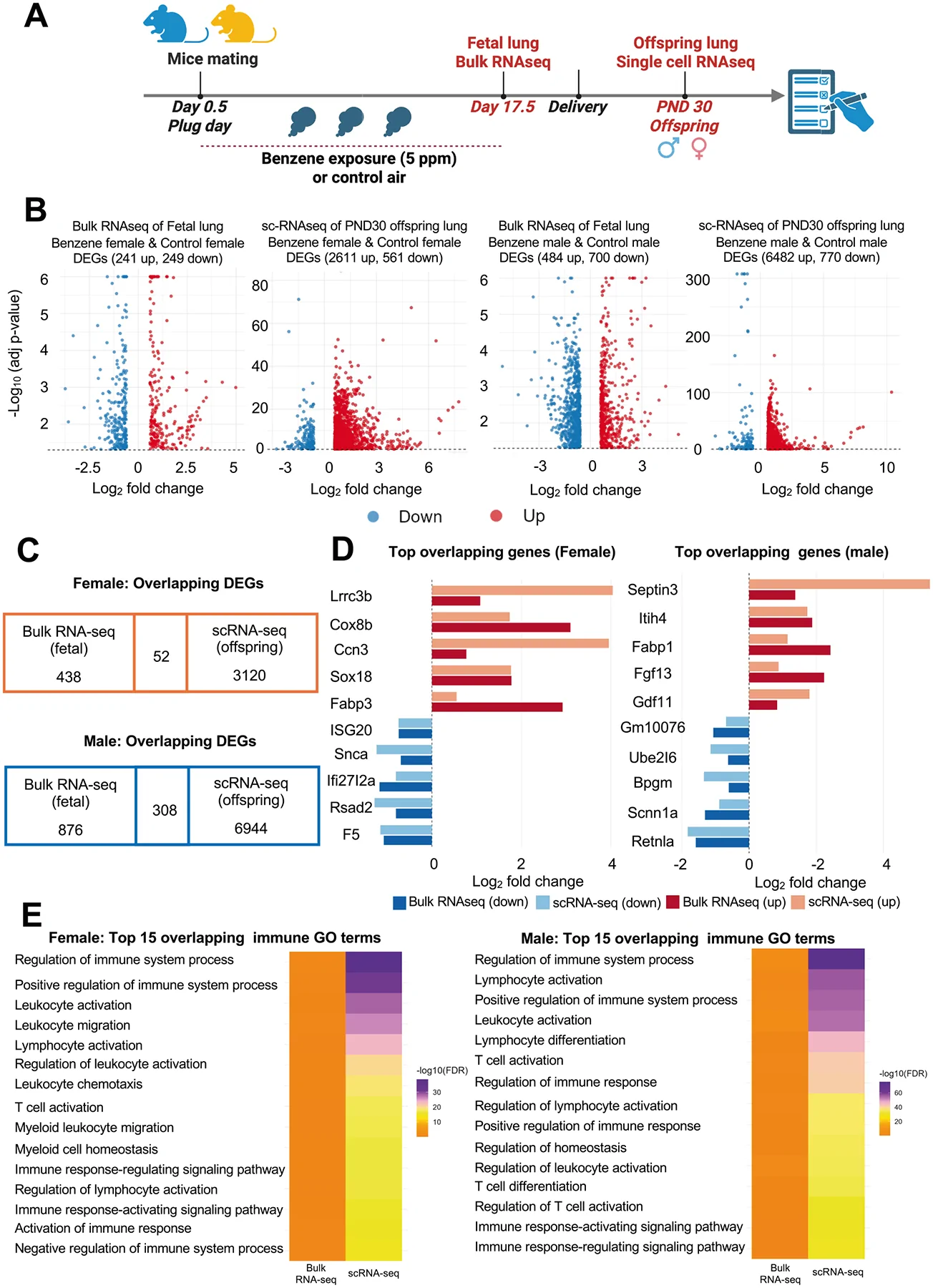
Persistent transcriptional reprogramming of the developing lung following gestational benzene exposure. (A) Schematic of the experimental design showing maternal benzene inhalation exposure (5 ppm, E0.5–E17.5). At E17.5, dams were transferred to a clean-air environment for delivery, and offspring were maintained under unexposed conditions until PND30 for scRNA-seq sequencing analysis. Created in BioRender. (B) Volcano plots showing significantly differentially expressed genes (DEGs; P < 0.05, |log_2_FC| > 0.25) identified from E17.5 fetal lung bulk RNA-seq and PND30 offspring pseudobulk scRNA-seq datasets in male and female lungs. (C) Venn diagrams showing the overlap of DEGs between E17.5 fetal lungs and PND30 offspring lungs within each sex. (D) Diverging bar charts showing the top shared up- and down-regulated genes in males and females, illustrating persistent transcriptional changes across developmental stages. (E) Gene Ontology (GO) enrichment analysis of biological processes filtered to the “immune system process” hierarchy. The heatmap shows the top ten significantly enriched GO terms (FDR < 0.05) that are common to both E17.5 fetal lung RNA-seq and PND30 pseudobulk scRNA-seq datasets within each sex.

Build on the observed sex-specific fetal lung inflammation, we next examined whether gestational benzene exposure induces transcriptional changes in the fetal lung that persist after birth. Plug-positive C57BL/6 dams were exposed to 5 ppm benzene or control air from E0.5 to E17.5. Fetal lungs were collected at E17.5 for bulk RNA-seq. In a parallel cohort, dams were transferred to clean air at E17.5 and allowed to deliver naturally. Offspring were maintained under clean conditions until PND30, when lung single-cell suspensions were prepared for scRNA-seq (Fig. 6A). We then compared prenatal bulk RNA-seq with postnatal pseudobulk profile from scRNA-seq datasets to identify persistent transcriptional signatures (FDR ≤ 0.05, |log_2_ FC| ≥ 0.25). Volcano plots revealed robust exposure-associated transcriptional changes in both fetal and offspring lungs of each sex (Fig. 6B).

To identify persistent reprogramming, we examined genes shared between fetal and postnatal lungs (Fig. 6C). In females, several top shared downregulated genes were classical ISGs, including *Isg20*, *Ifi27l2a* and *Rsad2 (Viperin)*, all of which contribute to antiviral immune regulation (Fig. 6D). In males, *Ube2l6*, *Scnn1a*, and *Retnla* were among the most differentially expressed overlap genes (Fig. 6D), representing pathways involved in ISGylation-depedent antiviral signaling, macrophage-mediated type-2 inflammation, and epithelial barrier maintenance, respectively.^33–35^ Finally, immune-related GO analysis of overlapping gene sets revealed persistent enrichment of immunological processes such as “regulation of immune system process,” “leukocyte activation,” and “lymphocyte activation” (Fig. 6E). Together, these data indicate that gestational benzene exposure establishes long-lasting, sex-specific transcriptional reprogramming of pulmonary immune networks from fetal development into postnatal life.

### Gestational benzene exposure drives pro-inflammatory reprogramming in offspring alveolar macrophages

**Fig. 7.**
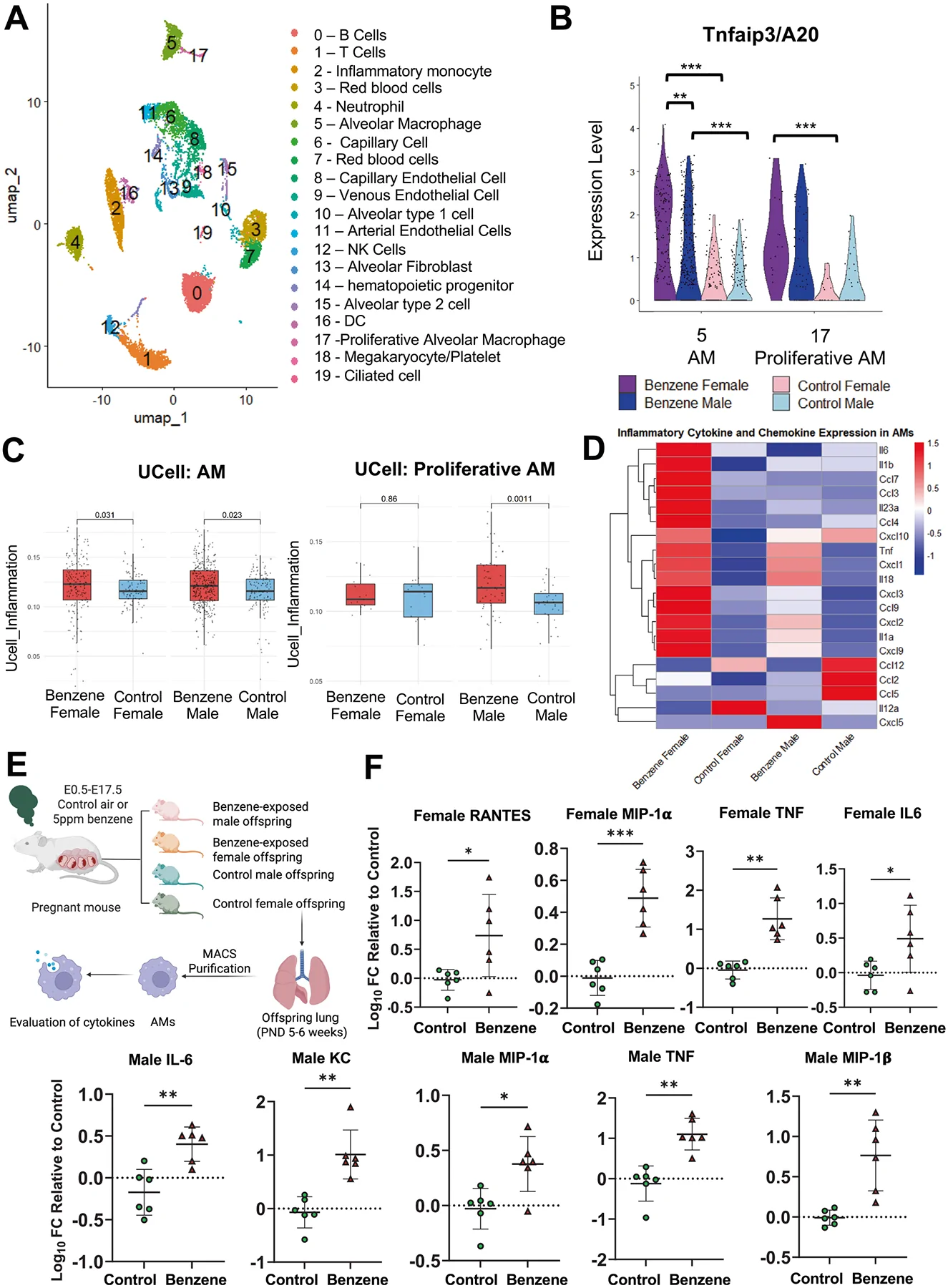
Persistent inflammatory priming of offspring alveolar macrophages following gestational benzene exposure. (A) UMAP of PND30 lungs showing annotated cell clusters identified by scRNA-seq. (B) Violin plots showing Tnfaip3 (A20) expression levels in AMs and proliferative AMs from male and female offspring. (C) Inflammation module scores for AMs and proliferative AMs calculated using UCell analysis. (D) Heatmap of relative expression of inflammatory cytokine genes in AMs from male and female offspring of control and benzene-exposed dams. (E) Experimental schematic of primary AM isolation and ex vivo analysis. Created with BioRender. Pregnant dams were exposed to benzene vapor (5 ppm) from E0.5–E17.5, then transferred to a clean-air environment for delivery. Offspring were maintained under unexposed conditions until 5–6 weeks of age. AMs were isolated from offspring lungs by MACS, plated for 24 h, and conditioned media were collected for cytokine analysis by Luminex. (F) Cytokines concentrations in AM conditioned media measured by Luminex and expressed as log_10_-transformed fold change relative to run- and sex-matched controls. For each group, n = 6 offspring were analyzed, representing 2 independent dams/litters with 3 offspring sampled from each dam/litter. Data are presented as mean ± SD; statistical comparisons were performed using unpaired *t* tests. *P <* 0.05 (*), P < 0.01 (**), P < 0.001 (***), P < 0.0001 (****).

To define the cellular populations underlying persistent immune alterations, we performed single-cell RNA sequencing on PND30 offspring lungs. Uniform Manifold Approximation and Projection (UMAP) analysis revealed the expected major lung cell types (Fig. 7A). Given prior DREAM and A20 dysregulation (Fig. 4), we assessed DREAM and A20 gene expression across cell populations. *Kcnip3* (DREAM) transcripts were most abundant in NK cells but were not altered by gestational benzene exposure (Supplementary Fig. S3A, B). In contrast, *Tnfaip3* (A20) transcripts were significantly increased by gestational benzene exposure in both alveolar macrophages (AMs) and the proliferative AM subset in offspring of both sexes (Fig. 7B), with a similar increase observed in neutrophils (Supplementary Fig. S4A).

Moreover, AMs from offspring of benzene-exposed dams exhibited significantly higher inflammation scores in both sexes compared with controls (Fig. 7C), while neutrophils showed an even more pronounced increase (Supplementary Fig. S4B). Heatmap analysis of canonical inflammatory genes in AMs revealed elevated expression of IL-6, IL-1β, IL-18, and CCL family members, with female AMs appearing more inflamed than males (Fig. 7D). Evaluation of other immune populations showed that neutrophils displayed a comparable induction of pro-inflammatory genes in both sexes (Supplementary Fig. S4C), whereas monocytes and dendritic cells exhibited selective downregulation of certain cytokines, such as CCL and CXCL family members (Supplementary Fig. S5). These findings indicate that gestational benzene exposure broadly alters pulmonary immune profiles, potentially affecting immune cell recruitment, chemotaxis, and functional responses during infection and inflammation.

To validate that gestational benzene exposure reprograms AMs toward a pro-inflammatory phenotype, we isolated AMs from PND 5–6-week-old offspring by MACS, and quantified cytokine secretion after 24 h in culture (Fig. 7E). Female AMs showed higher levels of RANTES, MIP-1α, TNF, and MIP-1β, whereas male AMs displayed significantly elevated IL-6, KC, MIP-1α, TNF, and MIP-1β (Fig. 7F). Together, these findings indicate that gestational benzene exposure reprograms AMs toward a persistent, sex-specific pro-inflammatory state.

### Gestational benzene exposure imprints sex-specific antimicrobial responsiveness in offspring alveolar macrophages

**Fig. 8.**
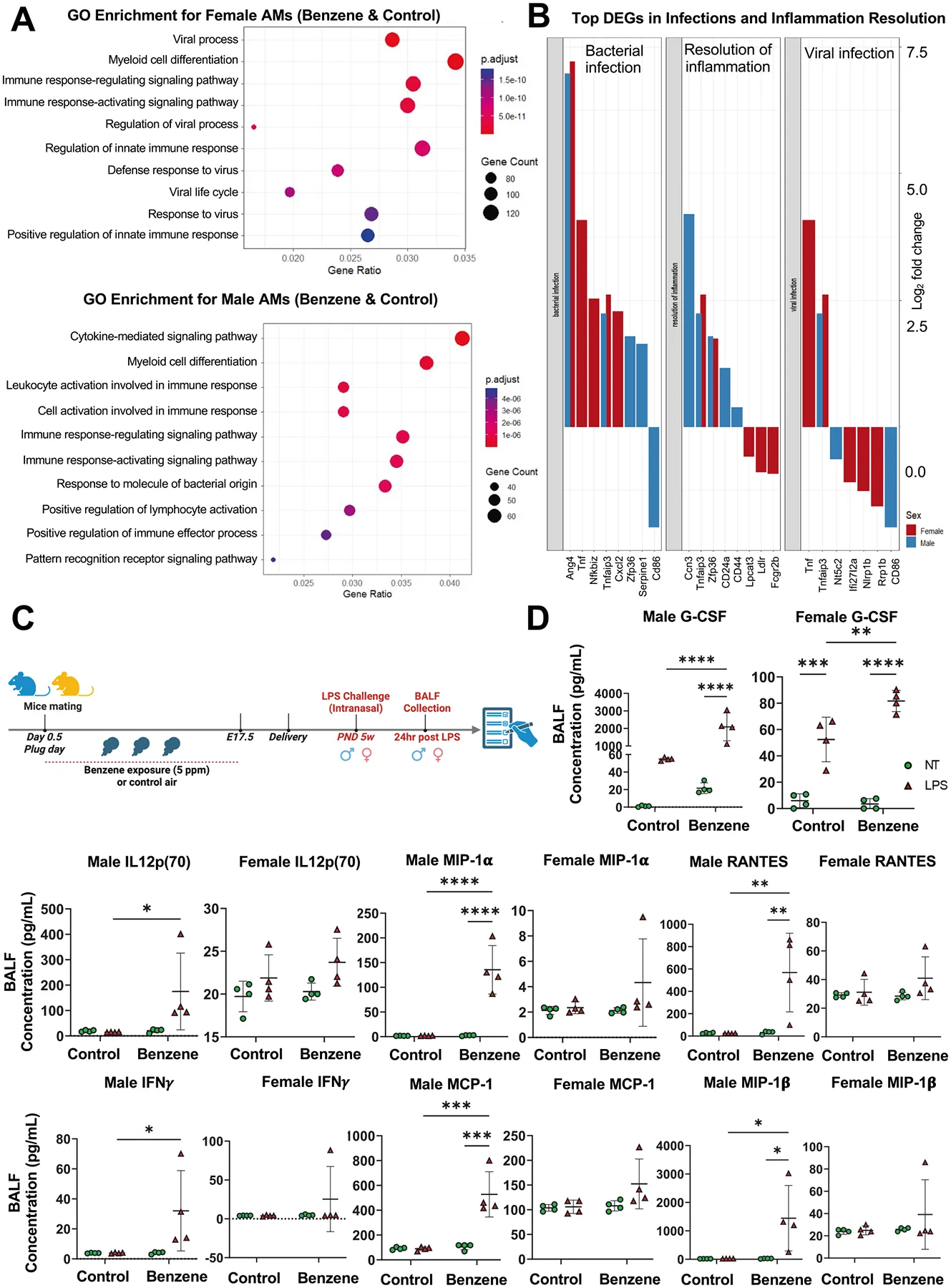
Sex-specific inflammatory reprogramming of alveolar macrophages and increased bacterial susceptibility in males following gestational benzene exposure. (A) Top enriched Gene Ontology (GO) biological processes in AMs from scRNA-seq of offspring from control or benzene-exposed dams, shown separately for males and females. (B) Top DEGs associated with bacterial infection, viral infection, and resolution of inflammation in AMs from male and female offspring. (C) Experimental schematic of postnatal intranasal LPS challenge. Created with BioRender. Pregnant dams were exposed to benzene vapor (5 ppm) from E0.5–E17.5, then transferred to a clean-air environment for delivery. Offspring were maintained under unexposed conditions until 5 weeks of age, then intranasally administered LPS (0.8 mg/kg). Mice were sacrificed 24 h later, and bronchoalveolar lavage fluid (BALF) was collected for analysis. (D) Cytokine concentrations in BALF from five-week-old offspring following LPS challenge, measured by Luminex and shown for males and females. For each group, n = 4 offspring were analyzed, representing 2 independent dams/litters with 2 offspring sampled from each dam/litter. Data are presented as mean ± SD; statistical comparisons were performed using two-way ANOVA with Tukey’s post hoc test where appropriate. *P <* 0.05 (*), P < 0.01 (**), P < 0.001 (***), P < 0.0001 (****).

To define persistent transcriptional reprogramming in offspring AMs, we conducted GO enrichment analyses separately by sex. Female AMs displayed strong enrichment of virus-associated pathways, including “viral process,” “regulation of viral process,” and “defense response to virus”. In contrast, male AMs exhibited enrichment of bacteria-related processes such as “response to molecule of bacterial origin” (Fig. 8A). These sex-dimorphic antimicrobial GO signatures support our findings that gestational benzene exposure predominantly affects viral control in female offspring (Figs. 2 and 3) and raise the possibility that male offspring may instead exhibit altered antibacterial responsiveness. We next examined the top DEGs associated with bacterial infection, viral infection, and resolution of inflammation. Notably, Tnfaip3 (A20) was upregulated in all three categories in both sexes (Fig. 8B), highlighting its central role in mediating gestational benzene–induced reprogramming of pulmonary immunity in both female and male offspring.

To test whether gestational benzene exposure predisposes male offspring to heightened bacterial responses, 5-week-old offspring were challenged intranasally with LPS (0.8 mg/kg). BALF cytokines were measured by Luminex 24 h post-challenge (Fig. 8C). Alveolar macrophages are the primary source of BALF cytokines under steady-state and inflammatory conditions, with recruited neutrophils and monocytes amplifying the response during inflammation.^36^ G-CSF was the only cytokine significantly elevated in both sexes from benzene-exposed dams following LPS challenge (Fig. 8D). Strikingly, male offspring from benzene-exposed dams exhibited significantly elevated IL-12(p70), MIP-1α, RANTES, IFN-γ, MCP-1, and MIP-1β compared with male controls, whereas female offspring showed no significant changes (Fig. 8D). These results indicate that gestational benzene exposure reprograms alveolar macrophages in a sex-specific manner, predisposing male offspring toward exaggerated responsiveness to bacterial stimuli.

## Discussion

In this study, we demonstrate that sex-specific regulation of antiviral immunity in the lung emerges during fetal development and that prenatal benzene exposure durably reprograms lung immunity in a sex-specific manner. We show that benzene metabolites accumulate within the placenta and fetal lung during gestation, coinciding with sex-dimorphic inflammatory signaling and immunological transcriptional remodeling of the developing fetal lung. These prenatal alterations persist into postnatal life, where female offspring exhibit enhanced viral control accompanied by excessive interferon-driven inflammation linked to disruption of the DREAM–A20 regulatory axis, whereas males display increased susceptibility to bacterial challenge. Single-cell analyses identify alveolar macrophages as a major affected innate immune population, exhibiting persistent inflammatory priming and sex-dependent functional responses. Together, these findings provide new insight into how gestational environmental toxicant exposure intersects with biological sex to impact long-term pulmonary immune trajectories.

Sex-specific differences in lung development are established in utero and are characterized by differential structural maturation between females and males.^37^ Human studies demonstrate accelerated lung maturation in female fetuses between mid- and late gestation,^38^ earlier functional pulmonary competence measured by lecithin–sphingomyelin ratio,^39^ and widespread sex-biased gene expression programs related to lung structural and functional development.^40^ Although these studies establish clear sexual dimorphism in lung maturation, direct evidence for sex-specific immune programming within the fetal lung remains limited. The identification of sex-biased autosomal microRNAs in human fetal lungs^41^ and sex-dependent immune dysregulation in animal models following prenatal stressors^13,42^ supports the concept that fetal immune development is intrinsically modulated by biological sex; however, most fetal lung transcriptomic studies pool samples irrespective of sex and focus primarily on structural maturation rather than immune signaling. Our findings extend this literature by demonstrating that immune pathways in the developing fetal lung are themselves sexually dimorphic and highly sensitive to environmental perturbation.

Within the Developmental Origins of Health and Disease framework, events occurring during gestation can exert lasting effects on pulmonary structure, immune function, and disease susceptibility.^6–8^ The fetal lung is particularly vulnerable, as immune progenitors seed the tissue early in gestation and are capable of sensing intrauterine inflammatory and environmental stimuli.^4,5^ Single-cell transcriptomic analysis revealed that prenatal benzene exposure induces persistent, sex-dependent reprogramming of alveolar macrophages characterized by inflammatory priming and altered cytokine responsiveness. This persistent phenotype may reflect durable developmental imprinting of tissue-resident alveolar macrophages, as these cells arise largely from fetal monocytes during the perinatal period and are subsequently maintained by local self-renewal with limited steady-state replacement by circulating monocytes.^25,43^ Thus, prenatal benzene exposure may establish stable functional changes in this macrophage pool, potentially through epigenetic or metabolic remodeling consistent with innate immune memory/trained immunity.^44^ Future chromatin accessibility, DNA methylation, or single-cell multi-omic studies will be needed to determine whether sex-specific epigenetic programs sustain inflammatory priming in the offspring lung. Moreover, such imprinting in lung-resident immune cells may shift baseline immune regulation, predisposing offspring lungs to amplified responses upon subsequent challenges. Future studies using alveolar macrophage depletion, adoptive transfer, or cell-specific perturbation approaches will be important to determine whether these reprogrammed macrophages directly mediate the altered interferon regulation and exaggerated inflammatory responses induced by prenatal benzene exposure. Furthermore, consistent with prior observations that maternal exposure to nitrogen dioxide, cigarette smoke, or alcohol induces sex-specific transcriptional responses in fetal lung tissue,^12–15^ we demonstrate that inhalational benzene exposure leads to accumulation of benzene metabolites in both the placenta and fetal lungs, coinciding with coordinated placenta–fetal inflammatory signaling in a sex-dependent manner. Importantly, these immune alterations occurred in the absence of significant fetal growth restriction or pregnancy loss, although prenatal benzene exposure was associated with reduced placental weight in male fetuses. The positive association between placental inflammation and fetal lung immune activation supports a model in which developmental programming may occur through both altered placenta-to-fetus signaling and direct delivery of toxicant metabolites to fetal organs.

Importantly, the transcriptional reprogramming observed in the fetal lung was not transient. Comparative analyses revealed overlapping gene expression signatures between fetal and postnatal stages, indicating persistent remodeling of immune regulatory pathways. Such sustained transcriptomic imprinting is consistent with previous reports that prenatal stressors produce long-term, sex-dependent alterations in pulmonary function and immune responsiveness.^16–18,45,46^ Functionally, this persistent reprogramming manifested as sex-specific postnatal host defense phenotypes. Female offspring from benzene-exposed dams displayed enhanced interferon-mediated antiviral responses accompanied by exaggerated inflammatory pathology, whereas males exhibited increased susceptibility to bacterial infection. However, these sex-specific differences cannot be attributed solely to prenatal benzene exposure, as postnatal lung and immune maturation are still ongoing at this early challenge stage and may progress differently between male and female mice.

Mechanistically, our findings suggest that altered regulation of the DREAM-A20 axis may contribute to female-biased interferon hyper-activation. DREAM (downstream regulatory element antagonist modulator) represses A20/TNFAIP3 expression,^31^ and A20 functions as a critical ubiquitin-editing enzyme that terminates NF-κB and type I interferon signaling, thereby maintaining immune homeostasis.^47^ Reduced DREAM expression was observed in female lungs both during fetal development and after birth, indicating persistent alteration of this regulatory pathway following gestational benzene exposure. In contrast, impaired A20 induction became apparent only upon postnatal viral challenge, suggesting that prenatal DREAM downregulation may be associated with a latent defect in interferon negative feedback that is unmasked by a secondary inflammatory stimulus. Together, these findings identify the DREAM-A20 pathway as one potential regulatory mechanism linking prenatal exposure and exaggerated postnatal immune responses. Although sex-dependent pulmonary immune imprinting has been attributed to hormonal influences,^38,39^ placental regulation,^19,20^ stress signalling,^21^ epigenetic modification,^22,23^ and early immune cell programming,^24,25^ these frameworks largely describe associations. Our data support a model in which prenatal perturbation of DREAM expression is associated with altered A20 induction and interferon dysregulation upon postnatal challenge, while future studies using alveolar macrophage-specific DREAM or A20 manipulation, including conditional knockout models, will be important to define the functional contribution of this pathway.

Collectively, our findings underscore in utero immune reprogramming as a critical determinant of long-term pulmonary immune health. By integrating developmental sexual dimorphism with environmentally induced transcriptomic remodeling and defined molecular checkpoint disruption, this study advances our understanding of how prenatal exposures shape persistent mucosal immune regulation. Recognition of these developmental “two-hit” interactions provides a framework for understanding sex-biased susceptibility to respiratory infection and inflammatory lung disease across the lifespan.

## Materials and Methods

### Mice

Male and female C57BL/6 mice (The Jackson Laboratory, 8–10 weeks old) were housed in a specific pathogen–free facility. Timed matings were established; vaginal plug detection was designated E0.5. Plug-positive females were randomly assigned to control air or continuous benzene (5 ppm) inhalation exposure (CH Technologies) from E0.5 to E17.5. Prenatal benzene exposure experiments were performed across independent exposure cohorts, and samples generated from these cohorts were allocated to multiple downstream analyses, including transcriptomic, immunologic, histologic, metabolite, and validation assays. On E17.5, maternal lung/liver, placenta, and fetal lung were collected; tissue portions were snap-frozen in liquid nitrogen within 20 min for metabolite analysis, with the remainder stored at − 80 °C. Placental and fetal tissues were collected from 2 to 3 independent dams/litters for each experimental group whenever possible, as indicated in the corresponding figure legends.

For viral challenge, pregnant dams delivered naturally. Pups were infected intraperitoneally with 65 PFU MHV68 at postnatal day 7 and euthanized 10 days later for serum, lung, liver, and spleen collection. This early-life challenge timepoint was selected to evaluate immune responses during a vulnerable developmental window in which the offspring lung and immune system are still maturing and may be particularly sensitive to prenatal environmental programming. A separate cohort was aged to PND30–40 for alveolar macrophage isolation and single-cell RNA sequencing. For postnatal offspring analyses, male and female pups were selected from 2 to 3 independent dams/litters whenever possible, and the number of offspring and independent dams/litters represented in each experiment is indicated in the corresponding figure legends. Fetal and placental sex was determined by qRT-PCR for Ddx3y. All experiments analyzed males and females separately. All animal procedures were approved by the Wayne State University Institutional Animal Care and Use Committee and conducted in accordance with National Institutes of Health guidelines.

### Virus infection and viral Quantification

MHV68 was propagated in NIH 3 T3 cells maintained in DMEM supplemented with 10% FBS. When complete cytopathic effect was observed, the supernatants were collected and filtered through a 0.45 μm pore filter. Viral titers were determined by plaque assay, and the virus was then aliquoted and stored at − 80 °C. For assessment of viral replication, total RNA was extracted from mouse tissues and viral transcripts were quantified by qPCR using primers targeting MHV68 genes ORF50, ORF65, and ORF74, with transcript levels normalized to GAPDH expression.

### RNA Extraction and Quantitative Real-Time PCR

Total RNA was extracted from mouse tissues using Trizol reagent according to the manufacturer’s instructions. RNA purity were assessed by A260/A280 ratio (≥1.8). One microgram of RNA was reverse transcribed into cDNA using the iScript cDNA Synthesis Kit (Bio-Rad, Hercules, CA, USA). Quantitative PCR was performed using iTaq Universal SYBR Green Supermix (Bio-Rad) with gene-specific primers (Supplementary Table S1). Reactions were run on the Bio-Rad CFX96 system (C1000 thermal cycler). Relative gene expression was calculated using the 2^−ΔΔCt^ method and normalized to housekeeping genes.

### Western blot

Tissues were homogenized in lysis buffer containing 1% Triton X-100 and protease inhibitors and clarified by centrifugation. Protein concentrations were determined using a BCA assay (Pierce, #23223, Rockford, IL). Equal amounts of protein (50 μg) were separated by SDS–PAGE and transferred to PVDF membranes. Membranes were blocked in 5% milk in PBS-T and incubated overnight at 4 °C with primary antibodies, followed by HRP-conjugated secondary antibodies. Signals were detected using enhanced chemiluminescence and imaged on an ImageQuant LAS 500 system (GE Healthcare). Band intensities were quantified using ImageJ (v1.49). Primary antibodies anti-A20 (Cell Signaling Technology [CST], #5630), anti-DREAM (Sigma, #SAB5200073), anti-TBK1 (CST, #3504), and anti-pTBK1 (CST, #5483) were used at 1:1,000. Anti-GAPDH (Sigma, #G8795) was used at 1:10,000. Peroxidase-conjugated anti-rabbit and anti-mouse IgG (CST, #7074 and #7076) were used at 1:10,000.

### Benzene metabolite analysis

Tissue samples were homogenized in methanol containing the internal standard N-Acetyl-S-phenyl(d5)-L-cysteine (PMA-d5) and subjected to phase separation. The aqueous fraction was diluted and analyzed by UHPLC–MS/MS using a C18 column coupled to a Thermo triple quadrupole mass spectrometer operating in multiple reaction monitoring (MRM) mode for detection of N-Acetyl-S-phenyl-L-cysteine (PMA) and PMA-d5. Quantification was performed using internal standard–normalized calibration curves generated from identically processed standards. The assay demonstrated linearity from 2–5000 pg, with a lower limit of detection of 1 pg.

### Histology

For morphological analysis, PND17 control and MHV68-infected mice were euthanized under isoflurane. Lungs were perfused via the left ventricle with 3 mL PBS containing 10 U/mL heparin, inflated with 4% paraformaldehyde (PFA) through the trachea, and fixed in 4% PFA overnight at 4 °C. Tissues were then ethanol-dehydrated, xylene-cleared, paraffin-embedded, sectioned (5 μm thick), and stained with hematoxylin and eosin (H&E). Images were acquired using an Echo Revolve microscope under consistent settings.

### Cytokine analysis

Cytokine concentrations were measured using the Bio-Plex Pro Mouse Cytokine 23-plex Assay (Bio-Rad, #60009RDPD). Assays were performed according to the manufacturer’s protocol. Data were acquired on a Luminex 200 system (Luminex, Austin, TX) and analyzed with xPONENT software (v4.3).

### Alveolar Macrophage isolation

Alveolar macrophages were isolated from mouse lungs using Anti-Siglec-F MicroBeads and LS columns (Miltenyi Biotec, #130–118–513) according to the manufacturer’s protocol. Lung single-cell suspensions were incubated with Anti-Siglec-F MicroBeads and subjected to magnetic separation, with a second column purification step to increase purity. Purity of the isolated fraction was assessed by flow cytometry, which confirmed that the Siglec-F^+^ population consisted predominantly of alveolar macrophages (~70–80%), with minimal contamination from other cell types and debris.

### Statistical analysis

Statistical analyses were performed using GraphPad Prism v8 (GraphPad Software). Data are presented as mean ± standard deviation (SD). Normality was assessed with the Shapiro–Wilk test. Data that did not follow a normal distribution were either transformed (log_10_) or analyzed using non-parametric tests. Homogeneity of variance was evaluated using Levene’s test. Comparisons between two groups were made using two-tailed Student’s t-tests. One-, two-, or three-way ANOVA was used as appropriate to assess main effects and interactions, followed by Tukey’s multiple-comparison test when significant. Correlation analyses between placental and fetal lung parameters were conducted within each exposure-by-sex group using Spearman’s rank correlation (two-tailed). A p value < 0.05 was considered statistically significant. For animal studies, each data point represents an individual offspring. Male and female offspring were selected from 2 to 3 independent dams/litters. The number of offspring and independent dams/litters included in each experiment is indicated in the corresponding figure legends.

### RNA sequencing and Bioinformatics analysis

Total RNA from fetal lungs was subjected to 3′ mRNA sequencing (Lexogen QuantSeq) at the Wayne State University Genomics Core. Libraries were sequenced on an Illumina NovaSeq platform. Reads were trimmed and aligned to the mouse reference genome (GRCm38/mm10), and gene-level counts were generated. Differential expression analysis was performed in R using edgeR with a negative binomial model. Genes with p < 0.05 and |log2 fold change| above the indicated threshold were considered significant. Functional enrichment analyses were performed using standard GO and KEGG frameworks (FDR < 0.05).

Single-cell RNA sequencing data were processed using Cell Ranger and analyzed in Seurat. Low-quality cells were excluded based on gene count and mitochondrial content thresholds. Data were normalized and subjected to PCA, clustering, and UMAP visualization. Differential expression was determined using the Wilcoxon rank-sum test with multiple-testing correction. Pathway analyses were conducted using clusterProfiler.

## Supplementary Material

1

## Data Availability

All data needed to evaluate the conclusions in the paper are present in the paper and/or the Supplementary Materials. The raw RNA sequencing data have been deposited in NCBI’s Sequence Read Archive (SRA) under accession number PRJNA1372098. Additional source data supporting the findings of this study are available from the corresponding author upon reasonable request.

## Appendix A. Supplementary data

Supplementary data to this article can be found online at https://doi.org/10.1016/j.mucimm.2026.100352.
