# Supplementary material for: Sex-specific in utero reprogramming of lung immunity
Source: Mucosal Immunol. Author manuscript; Available in PMC 2026 Aug 21. (PMC13495141; doi:10.1016/j.mucimm.2026.100352)
Supplement: Supplementary file 1 [file NIHMS2204573-supplement-1.docx]

Supplementary Materials for

**Sex-specific in utero reprogramming of lung immunity**

Anthony Maxwell *et al.*

*Corresponding author. Email: [candy.ding@wayne.edu](mailto:candy.ding@wayne.edu) (lead contact) and [gmor@med.wayne.edu](mailto:gmor@med.wayne.edu)

**This PDF file includes:**

Figs. S1 to S5

Tables S1

Fig. S1

**Figure S1. Enhanced IFIT gene expressions in female neonatal lungs following MHV68 infection after gestational benzene exposure.**

qRT-PCR analysis of *Ifit1, Ifit2* and *Ifit3* in PND17 offspring lungs following MHV68 infection, shown separately for females and males. Expression levels were normalized to *Ppia.* For each group, n=4-7 offspring were analyzed, representing at least 2 independent dams/litters with 2-3 offspring sampled from each dam/litter. Data are presented as mean ± SD; statistical comparisons were performed using two-way or three-way ANOVA with Tukey’s post hoc test where appropriate. *P* < 0.05 (**), P < 0.01 (**), P < 0.001 (****), *P < 0.0001 (*****).

Fig. S2

**Figure S2. Pregnancy outcomes following gestational benzene exposure at E17.5.**
Pregnancy outcomes were assessed in dams (n=5) exposed to control air or benzene during gestation. Implantation number, number of resorptions, and pregnancy loss rate were quantified per dam/litter. Placental and fetal weights were measured at E17.5 and analyzed separately for male and female fetuses. For implantation, resorption, and pregnancy loss analyses, each data point represents one dam/litter. For fetal and placental weight analyses, each data point represents one fetus or placenta, with at least 2-3 samples collected from 3 independent dams/litters. Data are presented as mean ± SD; statistical comparisons were performed using unpaired t-test or two-way ANOVA with Tukey’s post hoc test where appropriate. *P < 0.01 (**).*

Fig. S3.

**Figure S3: Kcnip3 (DREAM) expression across lung cell types at PND30.**

**(A)** Single-cell RNA-seq analysis of PND30 offspring lungs from control and benzene-exposed dams. Violin plots show *Kcnip3* expression across annotated cell clusters. Values represent log-normalized counts generated using the standard Seurat pipeline. **(B)** Violin plots of *Kcnip3* expression in NK cells, stratified by treatment and sex.

Fig. S4.

**Figure S4: Gestational benzene exposure reprograms offspring lung neutrophil toward a proinflammatory state.**

**(A)** Violin plots showing *Tnfaip3* expression in lung neutrophils from female and male offspring of control or benzene-exposed dams. **(B)** UCell inflammation scores for neutrophils from female and male offspring of control or benzene-exposed dams. **(C)** Heatmap showing expression of canonical inflammatory genes in neutrophils from female and male offspring of control or benzene-exposed dams.

Fig. S5.

**Figure S5. Gestational benzene exposure alters inflammatory gene signatures in offspring lung monocytes and dendritic cells.**
Heatmaps showing expression of canonical inflammatory gene signatures in lung monocytes and dendritic cells from female and male offspring of control or benzene-exposed dams.

**Table S1**

**Supplemental Table 1. Primer sequences for qRT-PCR.**

| **Gene** | **Forward (5'-3')** | **Reverse (5'-3')** |
| --- | --- | --- |
| Ifnα | GGATGTGACCTTCCTCAGACTC | ACCTTCTCCTGCGGGAATCCAA |
| Ifnβ | GCCTTTGCCATCCAAGAGATGC | ACACTGTCTGCTGGTGGAGTTC |
| Ifnγ | CAGCAACAGCAAGGCGAAAAAGG | TTTCCGCTTCCTGAGGCTGGAT |
| Ifit1 | TACAGGCTGGAGTGTGCTGAGA | CTCCACTTTCAGAGCCTTCGCA |
| Ifit2 | CGAACTACCGTCTGGATGACTG | CTTCAACCAGCGCCATTGCTTG |
| Ifit3 | GCTCAGGCTTACGTTGACAAGG | CTTTAGGCGTGTCCATCCTTCC |
| IL-6 | TACCACTTCACAAGTCGGAGGC | CTGCAAGTGCATCATCGTTGTTC |
| IL-1β | TGGACCTTCCAGGATGAGGACA | GTTCATCTCGGAGCCTGTAGTG |
| ORF50 | CAAAGTCCATAACAGGCATCC | AAATGCCTCAACTTCTCTGG |
| ORF65 | CCCAGAGCTCCATAACAAGC | AAATGCTCCAGAAGAGGAAGG |
| ORF74 | CTTAGAAAACTCATCATTGTCC | ACACAATAGCATAGATCCTGG |
| Kcnip3 (DREAM) | GCATACCACTGAGCAAGAGGGA | TGATGGCGCACCGTGGATAACT |
| Tnfaip3 (A20) | AGCAAGTGCAGGAAAGCTGGCT | GCTTTCGCAGAGGCAGTAACAG |
| DDX3Y | TGTTAGTTGCCACACCAGGACG | TGGTGGCATTGTGTCCTGCTCA |
| PPIA | CATACAGGTCCTGGCATCTTGTC | AGACCACATGCTTGCCATCCAG |
